# Efficacy and safety of low- and high-intensity focused ultrasound in glioblastoma: a systematic review of preclinical and clinical studies

**DOI:** 10.1038/s41416-025-03325-6

**Published:** 2026-01-08

**Authors:** Muteb Alrashidi, Febe Ferro, Anas Almohammadi, Nawal Hamed Alyoubi, Ghada Saleem Alsarheed, James Joseph, Sourav Banerjee

**Affiliations:** 1 Division of Cancer Research, School of Medicine, University of Dundee, Dundee, UK; 2 Department of Radiological Sciences, College of Applied Medical Sciences, Imam Abdulrahman Bin Faisal University, Dammam, Saudi Arabia; 3 Department of Biomedical Engineering, School of Science and Engineering, University of Dundee, Dundee, UK; 4 Healthcare Technology Assessment and Planning Administration, Healthcare Technology Management Sector, King Saud Medical City, Riyadh Saudi Arabia; 5 Department of Health Education, Al-Noor Specialist Hospital, Ministry of Health, Makkah, Saudi Arabia; 6 Student at Alyamamah University, Riyadh, Saudi Arabia

**Keywords:** CNS cancer, Cancer

## Abstract

**Background:**

Glioblastoma (GBM) is an aggressive brain tumour with a poor prognosis despite existing multimodal treatments, limited by the blood-brain barrier (BBB) and high recurrence rates. Focused ultrasound (FUS), encompassing low-intensity (LIFU) and high-intensity (HIFU) modalities, offers non-invasive approaches to enhance drug delivery and ablate tumour tissue, respectively. To systematically evaluate the efficacy and safety of LIFU-mediated BBB opening and HIFU-induced thermal ablation in GBM treatment, comparing preclinical and clinical outcomes.

**Methods:**

This review searched PubMed, Scopus, and Web of Science (August 2024) for studies on FUS in GBM. Preclinical and clinical studies reporting efficacy (tumour response, survival) and safety outcomes were included. Data were extracted using a standardised form, with quality assessed via SYRCLE (preclinical) and ROBINS-I (clinical) tools. Narrative synthesis was performed due to study heterogeneity.

**Results:**

From 1817 records, 40 studies (26 preclinical, 14 clinical with 139 patients) were included. LIFU enhanced delivery of chemotherapies, immunotherapies, and nanoparticles, reducing tumour growth and extending survival in preclinical models (e.g., 26–81.2 days vs. 18–30.4 days), with clinical trials showing progression-free survival of 2.5–4.11 months, overall survival of 10–14 months, and 100% one-year survival with temozolomide in a small cohort (*n* = 6). HIFU achieved ~70% tumour growth inhibition preclinically but only ~10% ablation clinically. Both modalities were safe, with no severe adverse events, only mild, transient effects like petechiae (LIFU) and warmth (HIFU).

**Conclusions:**

LIFU and HIFU are promising, complementary GBM treatments, with LIFU excelling in drug delivery and HIFU in ablation. Large-scale, multicentre randomised controlled trials with standardised parameters are needed to validate their efficacy and safety and optimise their integration into clinical practice.

**PROSPERO registration:**

CRD42024627213.

## Introduction

Glioblastoma (GBM) is the most aggressive primary brain tumour in adults, characterised by rapid infiltration, necrosis, a cold tumour microenvironment, and microvascular proliferation. GBM can develop as a primary tumour or through malignant transformation from lower-grade gliomas, often linked to isocitrate dehydrogenase mutations. Despite existing multimodal treatments (resection, radiotherapy, chemotherapy), median overall survival is 14–16 months, progression-free survival is 7–8 months, and 5-year survival is ~9.8%. Even with advances in surgical techniques, the diffuse nature of the disease poses a significant challenge during resection. The rapid and diffuse infiltration of glioma cells along white matter tracts and blood vessels renders complete surgical removal nearly impossible, contributing to near-universal recurrence [1, 2].

In the United States, the average annual age-adjusted incidence of primary brain and other central nervous system (CNS) tumours between 2016 and 2020 was 24.83 per 100,000 population (malignant: 6.94; non-malignant: 17.88). GBM accounted for 14.2% of all primary CNS tumours and 50.9% of malignant tumours. The five-year relative survival rate for malignant primary CNS tumours was 35.7% [3].

GBM exhibits a heterogeneous microenvironment with large populations of self-renewing, highly tumourigenic glioma stem cells that have been extensively studied and described [4]. Beyond stem-like cells, GBM includes diverse malignant cell states and non-neoplastic elements such as astrocytes, OPCs, endothelial cells, and pericytes that interact to drive tumour growth and resistance [5]. Further complexity is added by the high number of infiltrating immune cells, including microglia and monocyte-derived macrophages, which are co-opted into an immunosuppressive phenotype. This ‘cold’ immune milieu is marked by low T cell infiltration and deficient antigen presentation, limiting the efficacy of immunotherapeutic strategies [6]. The mostly intact blood-brain barrier (BBB) restricts systemic therapy penetration, and high recurrence rates due to the stem-like nature of the cancer cells exacerbate challenges.

Given the aggressive nature of GBM and the limitations of current treatment approaches, there is an urgent need for innovative therapeutic approaches to improve patient outcomes. One such promising technology is focused ultrasound (FUS), a non-invasive technique that uses ultrasonic energy to target and treat brain tumours with precision [7]. FUS can be applied at different intensities: low-intensity focused ultrasound (LIFU) enhances drug delivery by temporarily opening the BBB, allowing greater penetration of chemotherapeutic agents into the tumour [8], while high-intensity focused ultrasound (HIFU) generates thermal ablation to directly destroy tumour tissue through heat [9].

The mechanisms of FUS depend on the applied energy levels [7, 10]. LIFU transiently opens the BBB by inducing stable cavitation of intravenously administered microbubbles, a process where microbubbles oscillate without collapsing [11]. These forces temporarily open tight junctions between endothelial cells, enhancing drug delivery to the tumour while minimising systemic toxicity [12, 13]. In contrast, HIFU uses higher energy levels to produce rapid heating of targeted tissue, leading to protein denaturation and coagulative necrosis, effectively ablating tumour cells and reducing tumour volume [14, 15].

Building on these biophysical principles, this review aims to systematically evaluate the current evidence on FUS in GBM treatment. Specifically, we will compare the efficacy and safety profiles of LIFU-mediated BBB opening and HIFU-induced thermal ablation. By synthesising the available preclinical and clinical studies, we aim to offer evidence-based recommendations for the use of FUS in GBM treatment, identify optimal treatment parameters, and highlight research gaps to guide future work.

## Methods

### Registration and protocol

This systematic review was conducted in accordance with the Preferred Reporting Items for Systematic Reviews and Meta-Analyses (PRISMA) guidelines [16]. The review protocol was prospectively registered in the PROSPERO (CRD42024627213).

### Literature search

A comprehensive search was conducted on 11 August 2024 across PubMed, Scopus, and Web of Science using MeSH terms and keywords related to GBM and FUS (full strategy available in PROSPERO: CRD42024627213).

### Inclusion and exclusion criteria

Studies were included if they reported original data on FUS for GBM treatment in both clinical and preclinical settings. Eligible studies encompassed animal models with GBM or GBM-like tumours induced via cell implantation, genetic manipulation, or chemical methods, as well as clinical trials involving GBM patients. Interventions had to involve either low-intensity FUS used to transiently open the BBB for enhanced drug delivery or high-intensity FUS for thermal ablation of tumour tissue. Studies were required to provide measurable outcomes on efficacy (tumour response, survival metrics) or safety and be published in English.

Studies were excluded if they were non-original research (reviews, editorials, abstracts), not focused on GBM, combined FUS with other therapies without isolating its effects, or lacked clear ultrasound parameters or measurable outcomes.

### Data extraction

Data were extracted using a standardised form capturing study characteristics, intervention details, and efficacy and safety outcomes, with two reviewers independently extracting data and resolving discrepancies via discussion with a third reviewer.

### Quality assessment

The quality of the included studies was assessed using two established tools tailored to the specific study designs. For in vivo animal studies, the SYRCLE risk of bias tool [17] was employed due to its suitability for evaluating preclinical research. For clinical studies, the ROBINS-I tool [18] was used to assess the risk of bias in non-randomised studies. Quality assessments were independently performed by two reviewers. Visual illustrations of the quality assessment results were generated using the robvis [19] website.

## Results

### Study selection

**Fig. 1:**
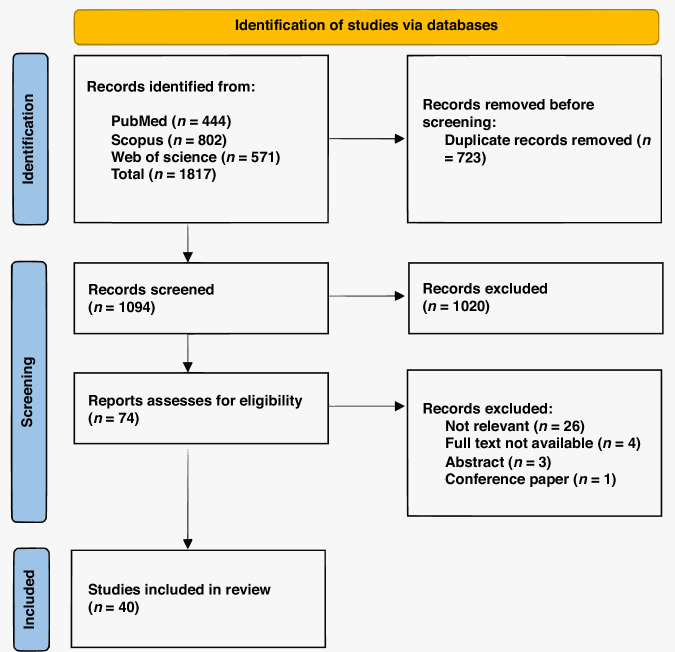
PRISMA flowchart of study assessment and selection. The flowchart outlines the identification, screening, and inclusion stages of the systematic review. A total of 40 studies were included after removing duplicates and screening records at the title/abstract and full-text levels. Reasons for exclusion at the full-text stage are specified. PRISMA Preferred Reporting Items for Systematic Reviews and Meta-Analyses.

Figure 1 depicts the selection process. A total of 1817 papers were initially identified. Following the removal of duplicates, 1094 records remained. The screening of titles and abstracts resulted in the exclusion of 1020 studies. A total of 74 full-text articles were evaluated for eligibility, with 34 papers excluded for reasons specified in the PRISMA flowchart. No additional papers were identified via reference searches. Finally, 40 studies [20–59] were included in this systematic review, meeting the established inclusion criteria.

### Study characteristics

Table 1 presents the characteristics of the 40 studies included in our review [20–59]. The table details study type, country, population or model, sample size, FUS modality, intervention specifics, efficacy outcomes, and safety outcomes for preclinical and clinical studies of FUS in GBM treatment. Of these, 26 preclinical studies used rodent models (e.g., GL261, U87, F98; 4–65 animals/group), testing chemotherapies (e.g., temozolomide, doxorubicin), immunotherapies, or sonodynamic therapy (SDT). The 14 clinical studies (phase I/IIa, one case report) enroled 139 patients with newly diagnosed or recurrent GBM. All identified clinical studies were conducted in high-income countries, including the USA, Canada, France, South Korea, Switzerland, and Taiwan, reflecting a limited but geographically varied distribution of research activity. Outcomes included survival, drug uptake, and mild, transient adverse events.

**Table 1:** Characteristics of 40 studies evaluating FUS in GBM treatment.

ID	Source	Study type	Country	Population/Model	Sample size	FUS modality	Intervention details	Efficacy outcomes	Safety outcomes
1	McDannold et al. [20]	Retrospective clinical cohort study	USA	Newly diagnosed glioblastoma (GBM) patients undergoing focused ultrasound (FUS) blood-brain barrier (BBB) opening.	Patients**:** *n* = 9 (38 sessions).	Low-intensity focused ultrasound (LIFU) delivered by a 220 kHz hemispherical phased array.	LIFU was performed before the temozolomide infusion, with continuous Definity microbubble infusion and subsonication-level power adjustment based on acoustic feedback.	Extensive BBB opening demonstrated on contrast-enhanced T1 magnetic resonance imaging (MRI).	Minimal petechiae were observed on susceptibility-weighted angiography MRI.
2	Fletcher et al. [21]	Preclinical animal study	USA	Healthy Sprague–Dawley rat brains and F98 glioma-bearing Fischer rats.	Mice: healthy, *n* = 19; tumour – survival study, *n* = 65; tumour acute study, *n* = 10.	LIFU delivered by a 220 kHz hemispherical phased array.	Microbubble (MB)-mediated FUS was delivered to 20 overlapping targets in the right striatum (5 ms bursts, ~1.1 Hz) with a 20 µl/kg bolus Definity injection; in the F98 model, identical FUS parameters were combined with radiation therapy (RT) (4, 8, or 15 Gy) with real-time harmonic feedback control.	In healthy rat brains, FUS + RT (8 & 15 Gy) produced persistent ablated lesions on MRI/histology. In the F98 model, FUS + RT at 4 Gy reduced tumour volumes by 45–57% and increased apoptosis and ceramide levels compared with FUS or RT alone.	In healthy rats, FUS alone produced transient oedema visible on T2-weighted MRI (resolved by day 17) without neurological deficits; histology showed no tissue damage in 4/5 rats and local scarring in 1/5.
3	Carpentier et al. [22]	Prospective, Phase I/II clinical trial. NCT03744026	France and USA	Recurrent GBM patients.	Patients: *n* = 33 (90 sonications).	LIFU with a nine-emitter implantable device (1 MHz, 10 mm emitters).	Nine-emitter device implanted during resection; sonication every 4 weeks with carboplatin; Phase I: escalating emitters (3, 6, 9); Phase II: 9 emitters; sonication parameters: 1.03 MPa, 25-ms pulses, 0.5 Hz, 270 s with Definity microbubbles.	BBB opening in 90% of emitters with grade 2-3 permeability; Progression-free survival (PFS): 2.5-3.1 months (Cohort C: 2.5, Cohort D: 3.1); Overall survival (OS): 12.0 months overall, Cohort D 14.0 months; Cohort D lower tumour growth rate (0.54 vs 2.31 mL/month in Cohort C).	No dose-limiting toxicities; Grade 3 Adverse events (AEs): pre-syncope (*n* = 3), fatigue (*n* = 1), wound infection (*n* = 2), pain (*n* = 7); lower-grade Treatment-emergent AEs: scalp pain, nausea, dizziness, headache, transient aphasia, blurred vision; neutropenia in 2 patients; new <5-mm hypointense regions in 11% of treatments (not clinically significant).
4	Bhoopathi et al. [23]	Preclinical animal study	USA	Human GBM xenografts (GBM6, MDA-GSC-8-11) and murine CT-2A glioma in nude or C57BL/6 mice.	Mice: *n* = 5–10 per group.	LIFU with a 1 MHz frequency.	Double microbubble (DMB) approach: intravenous (IV) empty MBs + FUS (1 min, 3.5 mV, 10 dB) to open BBB, followed by IV Ad-loaded MBs + FUS; Ads: Ad.5/3-CTV (oncolytic, IL-24), Ad.5/3-Luc, TCTV, Ad.5-MDA-7-Luc; some groups received TMZ (30 mg/kg IP, 5 doses); single or triple FUS-DMB treatments.	Reduced tumour growth via bioluminescence imaging; survival extended with FUS-DMB-CTV (e.g., GBM6: ~50% longer vs. control, p < 0.05; triple treatment further enhanced); equivalent to IC CTV injection; TMZ combination further increased survival; increased apoptosis (TUNEL + ), decreased proliferation (Ki-67-).	Transient BBB opening (S100β peak at 30 min, normal by 4 h); no behavioural changes (open field/rotarod tests at 4 h and 1 week); no short- or long-term toxicity; no systemic effects on weight or blood profile.
5	Arrieta et al. [24]	Translational (preclinical animal study & clinical pilot study). Related trials: NCT04528680; NCT05864534.	USA	Preclinical: GL261/CT-2A glioma-bearing C57BL/6 mice. Clinical: Recurrent GBM patients.	Mice: *n* = 3–10 per group. Patients: *n* = 4.	LIFU with a 1 MHz frequency (preclinical) and a nine-emitter implantable device (clinical).	Preclinical: LIFU with IV DOX and/or anti-PD-1 on days 7, 14 (DOX) and 7, 10, 14, 17 (anti-PD-1); sonication: 60 s, ~0.3 MPa with microbubbles. Clinical: LIFU with IV liposomal doxorubicin (DOX, 30 mg) and anti-PD-1 (pembrolizumab, 200 mg) via implantable device	Preclinical: 3.9-fold DOX, 6-fold anti-PD-1 increase; prolonged survival in mice with long-term survivors; enhanced immune activation (IFN-γ, MHC I/II upregulation). Clinical: 2-fold increase in DOX and anti-PD-1 brain concentration.	Preclinical: no obvious toxicity, normal body weight/histology, no neurological deficits. Clinical: well-tolerated, no severe toxicities.
6	Zhang et al. [25]	Preclinical animal study	USA	GL26 glioma-bearing Ccr2RFP/wtCx3cr1GFP/wt mice.	Mice – histology: *n* = 30 (4 groups, *n* = 5 each); flow cytometry: *n* = 10 (2 groups, *n* = 5 each) + 3 naïve.	LIFU with a 1.5 MHz eight-element annular array transducer.	MRI-guided LIFU with IV Definity microbubbles; 1–3 sessions every other day; acoustic pressure ~0.5 MPa; targeted at the tumour region; no adjunct drugs.	Increased infiltration of CX3CR1+ and CCR2+ TAMs after 2–3 sessions; higher monocytes and CD80+ pro-inflammatory TAMs/microglia (*p* < 0.05).	No micro-haemorrhages or oedema on MRI post-LIFU; transient and reversible BBB opening; no gross damage.
7	Sun et al. [26]	Preclinical animal study	USA	GL261 glioma-bearing C57BL/6J mice.	Mice – survival: *n* = 45 (Control *n* = 9, R2 *n* = 9, R2 + FUS *n* = 10, R15 *n* = 8, R15 + FUS *n* = 9); delivery: *n* = 11 (R2 *n* = 4, R15 *n* = 7); histology: *n* = 4.	LIFU with a 690 kHz spherically curved transducer.	LIFU (10 ms bursts, 4 Hz, 100 s, 0.32 MPa) with IV microbubbles; two sessions (days 14 and 20 post-tumour inoculation); IV HA-based DOX-HA-CPT conjugates; targeted at striatum tumours.	Tumour suppression via BLI (significant with R15 + FUS, *p* < 0.05); median survival 28 days (R15 + FUS) vs. 24 days (control); increased apoptosis (cleaved caspase-3) and T-cell infiltration (CD3 + , CD8 + ).	Transient BBB opening confirmed by T1-weighted MRI; no overt toxicity (e.g., no reported haemorrhages, oedema, or neurological deficits); safe in healthy mice for delivery study.
8	Sonabend et al. [27]	Prospective, Phase I clinical trial. NCT04528680	USA	Recurrent GBM.	Patients: *n* = 17 (68 sonications).	LIFU with a 1 MHz frequency.	LIFU (1 MHz, 4.5 min, 1.03 MPa, 25-ms pulses, 0.5 Hz) via a nine-emitter implantable device; IV microbubbles (perflutren, 10 µL/kg); IV albumin-bound paclitaxel (40–260 mg/m²) every 3 weeks, up to 6 cycles; targeted at peritumoural brain.	BBB opening in 3.5–20.9 mL (median 12.6 mL) of peritumoural brain; 3.7-fold increase in paclitaxel brain concentration (0.139 µM vs. 0.037 µM, p < 0.0001); median PFS 2.9 months (95% CI: 2.7–4.6); median OS 11 months (95% CI: 7.95–not reached).	Transient grade 1–2 headache (71%), paraesthesia (12%), weakness (24%), dysphasia (12%), dysarthria (12%), dysaesthesia (18%), blurred vision (29%); grade 3 encephalopathy (1 patient, 8%) at 260 mg/m², resolved in 1–2 days; grade 2 peripheral neuropathy (1 patient); grade 3–4 neutropenia (47%), leukopenia (29%), hypertension (29%); no treatment-related deaths.
9	Porret et al. [28]	Preclinical animal study	France	U251 glioma-bearing NMRI nude mice.	Mice – imaging: *n* = 12 (FUS vs non-FUS); survival: *n* = 28–44 (*n* = 7–11 per group).	LIFU with a 1.5 MHz focused transducer.	LIFU (430 kPa, 127 s, 69% duty cycle) with IV microbubbles (100 µL); zig-zag scan over 6 × 6 × 7 mm region; IV 89Zr-cetuximab (CTX, 40 mg/kg) for imaging (one session) or survival (5 sessions, twice weekly); targeted at striatum tumour.	Early CTX extravasation increased with LIFU ( ≤ 4 h); tumour uptake was ~5% of the injected dose at 24–72 h (no difference vs. non-FUS); median survival was 42 days (CTX or CTX + FUS) vs. 29 days (control); and mean survival with CTX + FUS was 39.82 days vs. 34.90 days (CTX alone, p = 0.0097 vs. control).	Transient BBB/BTB opening via T2 MRI; no brain damage; transient glial activation (TSPO-PET peak at 48 h, low impact).
10	He et al. [29]	Preclinical animal study	China	GL261 glioma-bearing C57BL/6 mice.	Mice – efficacy: *n* = 9 (3 groups, *n* = 3 each).	High-intensity focused ultrasound (HIFU) with a 1.1 MHz frequency.	HIFU (1 V, 4 min/session, 7 sessions every other day over 14 days) with IV FeDOX@cellMBs (SPIO + DOX); magnetic targeting (0.48 T, 3 h post-sonication); targeted at right striatum tumour.	Reduced tumour growth via BLI (p < 0.05 vs. PBS); increased brain accumulation (12–24 h) vs. FeDOX+cellMBs (p < 0.01); decreased proliferation (Ki67-, p < 0.05) and increased apoptosis (TUNEL + , p < 0.01).	No significant damage to brain, heart, liver, spleen, lungs, or kidneys (H&E); stable in vivo up to 24 h; no overt toxicity.
11	Chevaleyre et al. [30]	Preclinical animal study	France	Orthotopic GL261-GFP glioma in female C57BL/6 mice.	Mice: *n* = 24.	LIFU with a 1.5 MHz focused transducer.	LIFU (420 kPa, 127 s, 69% duty cycle) with IV microbubbles (50 µL); raster scan over 6 × 6 mm for near-whole-brain BBB opening; IV 89Zr-labelled anti-Programmed Death-Ligand 1 (PD-L1) IgG (C4 or C4Fc-MUT) 1–2 min post-FUS; targeted at the whole brain (tumours in the striatum).	Increased early and overall tumour/brain uptake of radioligands (p < 0.05 vs. sham); enabled PD-L1 imaging; C4Fc-MUT showed faster clearance and earlier tumour contrast (max tumour-to-contralateral ratio ~22 h).	No acute injury or structural damage (H&E); transient BBB opening (closes in 10–15 min); no neurological issues, weight loss, or long-term damage.
12	Chen et al. [31]	Translational (preclinical animal study & prospective pilot study). NCT04988750	Taiwan	Preclinical: GL261 glioma in male C57BL/6 mice. Clinical: Recurrent high-grade glioma patients.	Mice: *n* = 23 (4 groups). Patients: *n* = 6.	LIFU with a 500 kHz frequency.	Preclinical: LIFU (MI 0.4–0.56, 1% duty cycle, 120 s) with IV microbubbles (7 µL), whole-brain RT (2 or 5 Gy), targeted at striatum tumour. Clinical: LIFU (MI ≤ 0.68) with IV microbubbles (0.1 mL/kg), re-RT (30–40 Gy/10 fractions or 21–27 Gy/3 fractions), targeted at recurrent tumours.	Preclinical: RT-FUS (2 Gy) improved survival (p < 0.05) and reduced tumour volumes vs. RT alone. Clinical: Objective response rate 16.7%, median PFS ~ 97.5 days, 3/6 patients with stable disease >5 months.	Preclinical: No major safety signals. Clinical: No FUS-related AEs; 1/6 patients had grade 3 radiation necrosis (attributed to re-RT).
13	Lee et al. [32]	Preclinical animal study	USA	Female C57BL/6J mice with GL261 glioma.	Mice: *n* = 63.	LIFU with a 1.64 MHz frequency.	LIFU (1.64 MHz, 10 ms pulses, 1 Hz, ~2 min/target) with IV microbubbles (Definity) and closed-loop cavitation control; IV anti-PD1 or IgG control post-FUS; targeted at the tumour region.	Prolonged survival with LIFU + anti-PD1 vs. anti-PD1 alone (p < 0.05); 1 mouse achieved complete remission and resisted rechallenge; increased anti-PD1 penetration (2.5×), proinflammatory macrophages (CD64 + ), and memory T cells (CD69 + CD103 + CD8 + ).	Closed-loop control suppressed inertial cavitation; no major AEs; minimal inflammatory infiltration in the healthy brain.
14	Yang et al. [33]	Preclinical animal study	China	NOD-SCID mice with orthotopic T98G GBM.	Mice – survival: *n* = 25 (5 groups, *n *= 5 each); MRI: *n* = 15 (5 groups, *n* = 3 each); other sub-studies: group sizes not stated.	LIFU with a custom transducer.	LIFU (1.84 W, 3–5 min, optimised to 3 min) with IV lipid-polymer hybrid nanoparticles (LPHNs-cRGD) carrying CRISPR/Cas9 plasmids (pCas9/O [6]-methylguanine-DNA methyltransferase [MGMT]) and IV microbubbles (4 × 10^6); IV temozolomide (TMZ, 50 mg/kg/day ×5); targeted at right striatum tumour.	Reduced tumour growth (MRI, p < 0.05); median survival 43 days (LIFU + LPHNs-cRGD + TMZ) vs. 22–30 days (controls); MGMT knockdown restored TMZ sensitivity.	No significant tissue damage or haemorrhage at optimised parameters; no organ pathology (H&E: heart, liver, spleen, lungs, kidneys).
15	Wei et al. [34]	Preclinical animal study	USA	Orthotopic MGPP3 glioma in male B6(Cg)-Tyrc-2J/J mice.	Mice – survival: *n* = 44 (Control *n* = 13, Etoposide *n* = 13, FUS *n *= 7, FUS + Etoposide *n* = 11); PK/tumour growth: *n* = 6 per group.	LIFU with a 1.5 MHz frequency.	LIFU (1.5 MHz, 5 Hz pulse repetition frequency [PRF], 1 ms pulse, 0.7 MPa) with IV microbubbles (1 µL/g) and IP etoposide (5 mg/kg) on days 7 and 14; four-spot grid targeting tumour.	Reduced tumour growth (~45% vs. control, p < 0.05); median survival 25 days (FUS + Etoposide) vs. 19 days (controls, p < 0.01); 8-fold increase in tumour etoposide concentration.	No haemorrhage or tissue damage on MRI/histology; mostly stable cavitation; well-tolerated.
16	Sabbagh et al. [35]	Preclinical animal study	USA	GL261 and QPP4 gliomas in C57BL/6 mice; EGFRvIII–U87 in NSG mice.	Mice: *n* = 3–10 per group.	LIFU with a 1 MHz frequency.	LIFU (1 MHz, 1 Hz PRF, 25,000 cycles, 0.3 MPa, 120 s) with IV microbubbles (200 µL) and IV immunotherapy (anti-PD1, CAR T cells, or CXCL10 APC).	Anti-PD1 + LIFU: ~58 days survival vs. ~39 days (anti-PD1 alone); CAR T + LIFU: >80 days vs. 35 days (p < 0.05); CXCL10 APC + LIFU: 34 days vs. 24–28 days (p < 0.05).	No neurological deficits or significant toxicity; minimal immune changes from LIFU alone.
17	Park et al. [36]	Prospective clinical trial. NCT03712293	South Korea	GBM patients post-resection and chemoradiation.	Patients: *n* = 6 (145 treatment sessions).	LIFU with a 220 kHz frequency.	LIFU (220 kHz, 90-s sonications, 3 × 3 subspot grid, 3-mm spacing, ~2.65 sonications/target) with IV microbubbles (perflutren, 4 µL/kg bolus, max 20 µL/kg); oral TMZ (150–200 mg/m², 5 days/cycle) on day 1 or 2 of 6 monthly cycles; targeted at peritumoural white matter within 2 cm of the resection margin.	BBB opening in 92.4% of targets (134/145); radiologically stable disease in 5/6 patients during 6 cycles; 1/6 withdrew after 3 cycles.	No FUS-related haemorrhage, oedema, or neurological deficits; 1 TMZ-related grade 2 haematological AE; 1 pseudoprogression with transient weakness (resolved); safe across 6 cycles.
18	Molotkov et al. [37]	Preclinical animal study	USA	C57BL/6 mice.	Mice: *n* = 4 per group.	HIFU with a 1.5 MHz frequency.	HIFU (1.5 MHz, 0.7 MPa, 1 Hz PRF, 10 ms bursts) with IV microbubbles (4.5 × 10^7 in 0.5 mL DEFINITY®) and IV radiotracers ([11 C] topotecan or [18 F] FSPG); targeted at the right cerebral hemisphere.	Increased [11 C] topotecan uptake (~2.1%ID/g vs. 0.88%ID/g, p < 0.05) and a 2.2-fold higher volume of distribution confirmed regional BBB opening with.	No significant AEs were reported; BBB opening was confirmed without haemorrhage or major disruption.
19	Meng et al. [38]	Prospective, Phase I clinical trial. NCT03616860	Canada	GBM patients post-resection and chemoradiation.	Patients: *n* = 9 (a total of 38 sonications performed across treatment cycles).	LIFU with a 220 kHz frequency.	LIFU (220 kHz, 111 ± 39 min, 2.5-mm spaced sonication points) via hemispherical array device; IV microbubbles (Definity); oral temozolomide (dose per neuro-oncologist) 30 min prior; targeted at non-enhancing tumour and 1-cm peritumoural margin; monthly with temozolomide cycles.	BBB opening in 7.8 ± 6.0 cm³ (range 0.8–23.1 cm³); 2.6 ± 1.2-fold increase in plasma cfDNA (P < 0.01); 3.2 ± 1.9-fold increase in neurone-derived EVs (P < 0.01); 1.4 ± 0.2-fold increase in S100b (P < 0.01); 2–3-fold increase in isocitrate dehydrogenase 1 R132H mutant copies in 1 patient.	No serious AEs; well-tolerated, no FUS-related toxicities reported.
20	Liao et al. [39]	Preclinical animal study	Taiwan	Orthotopic C6-luciferase glioma in male Sprague-Dawley rats.	Rats: *n* = 55.	Shockwave (SW), PiezoWave device.	SW (~0.21 mJ/mm², 5 Hz, 200 pulses, ~40 s) with IV doxorubicin (3 mg/kg) on days 7, 9, and 13; targeted at the tumour-bearing hemisphere; no microbubbles.	Reduced tumour size (BLI, p < 0.05); median time to humane endpoint: 26 days (Dox + SW) vs. 18 days (control, p = 0.015); increased doxorubicin concentration (~400 ng/g vs. ~150–200 ng/g, p < 0.05).	No major haemorrhage or tissue damage (H&E, Nissl); minimal cavitation effects; potential risk of tumour migration noted.
21	Chen et al. [40]	Translational (preclinical animal study & prospective, phase I pilot study). NCT03626896	Taiwan	Preclinical: C6 glioma. Clinical: Patients with recurrent GBM.	Rats: *n* = 20. Patients: *n* = 6.	LIFU with a 500 kHz frequency.	Preclinical: Single-session LIFU (MI 0.63 or 0.81, 120 s) with microbubbles, targeting tumour. Clinical: Single-session LIFU (MI 0.48–0.68, 120 s/spot, 9 spots) with IV microbubbles (0.1 mL/kg), targeting the peritumoural region.	Preclinical: 0.81 MI increased CD4 + T-cell infiltration (p = 0.043) at day 7. Clinical: Dose-dependent BBB opening (0.58–0.68 MI robust).	Preclinical: No significant safety issues reported. Clinical: No FUS-related AEs, BBB closed by 24 h.
22	Chan et al. [41]	Preclinical animal study	Taiwan	D54MG glioma-bearing NOD-SCID mice.	Mice: *n* = 4–5 per group.	HIFU with a 7 MHz frequency.	HIFU (7 MHz) with IV Dox-FePt@NB-Tf nanobubbles (10 mg/kg, 2–3 doses on days 21, 25, and 29) and external magnetic guidance; targeted at frontal lobe tumours.	Subcutaneous: ~70% tumour growth inhibition; Orthotopic: reduced tumour size (H&E, MRI), improved survival (p < 0.05), enhanced T2 MRI contrast.	No major organ toxicity (H&E); minimal off-target effects; no haemorrhage reported.
23	Wang et al. [42]	Preclinical animal study	USA	C57BL/6 mice.	Mice: *n* = 4–5 per group.	LIFU with a ~ 0.4–0.6 MHz frequency.	LIFU (0.72 MPa) with IV microbubbles, single session targeting striatum in both hemispheres; post-RT (30 Gy in 5 fractions) at 2 days (acute) or 31 days (chronic).	BBB opening was verified (DCE-MRI, Ktrans); the acute group showed a trend of higher Gadolinium uptake in the irradiated hemisphere (p = 0.072).	No haemorrhage or necrosis (H&E); well-tolerated with no additional damage from FUS.
24	Park et al. [43]	Prospective clinical trial. NCT03712293	South Korea	Patients with newly diagnosed GBM.	Patients: *n* = 6.	LIFU with a 220 kHz frequency.	LIFU (220 kHz, ~210 s/target, 4.4 targets/session) with IV microbubbles, 6 monthly sessions targeting the peritumoural region, combined with TMZ (150–200 mg/m², 5 days/cycle).	1-year survival: 100%; 4/6 no recurrence, 2/6 recurred at 11 and 16 months; potential PFS extension.	No severe FUS-related AEs; 1 pseudoprogression with transient weakness (resolved); safe across 6 sessions.
25	McDannold et al. [44]	Preclinical animal study	USA	Healthy Sprague-Dawley rats; F98 glioma in Fischer rats.	Rats – safety: *n* = 15 (FUS-only *n* = 5, FUS + IN *n* = 5, IN-only *n* = 5); drug study: *n* = 16; tumour study: *n* = 16.	LIFU with 230 kHz frequency.	LIFU (230 kHz, 5 ms bursts, 1.1 Hz PRF/target, 55 s/sonication, 36 foci) with IV microbubbles (Definity, 10 μL/kg) ± IV Irinotecan (10–20 mg/kg), up to 3 weekly sessions; targeted at the right hemisphere or tumour + margin.	BBB opening in 98% of targets; irinotecan uptake increased (p < 0.01), SN-38 low (<50% detectable); no tumour growth inhibition or survival benefit in the F98 model (p > 0.05).	Minimal damage (rare petechiae; small scars in 4/10 rats); 1 FUS + IN death (hypothermia due to procedural error, not treatment-related); feedback control limited inertial cavitation (<1% wideband).
26	Curley et al. [45]	Preclinical animal study	USA	Athymic nude mice with U87 glioma; C57BL/6 mice with B16F1ova melanoma.	Mice: *n* = 4–8 per group.	LIFU with a 1.1 MHz frequency.	LIFU (1.1 MHz, 0.45–0.55 MPa, 10-ms bursts, 0.5% duty cycle, 2 min/spot, 8-spot grid) with IV microbubbles (1×10^5/g) and plasmid BPNs (1 μg/g); targeted at the right striatum tumour.	~4-fold increase in transgene expression (p < 0.05); 2-fold increase in interstitial flow velocity (p < 0.05); >2-fold increase in transfection volume (p = 0.0008).	No significant inertial cavitation or overt tissue damage (histology from prior studies); safe at 0.45–0.55 MPa.
27	Brighi et al. [46]	Preclinical animal study	Australia	NOD/SCID mice with orthotopic WK1 PDX glioma.	Mice: *n* = 14 (control *n *= 6, FUS *n* = 8).	LIFU with a 1.1 MHz frequency.	LIFU (1.1 MHz, 0.85 MPa, 10-ms bursts, 120 s/spot, 10–20 spots) with IV microbubbles (Definity) and radiolabelled anti-EphA2 antibody; targeted at nonenhancing tumour regions.	Increased antibody uptake in the tumour (p < 0.05, r = 0.86 with BBB opening volume); no tumour control assessed.	No haemorrhage or gross damage (H&E); localised astrogliosis and microgliosis (GFAP + , Iba1 + ).
28	Yoshida et al. [47]	Preclinical animal study	Japan	Fischer 344 rats with orthotopic F98 glioma.	Rats: *n *= 4–6 per group.	LIFU with a 220 kHz frequency.	LIFU (220 kHz, 5000 J total, 18 W, 30 s × 10, 100% duty cycle, ≤42 °C) with IP 5-ALA (100 mg/kg); targeted at right hemisphere tumour.	Tumour growth reduced at day 16 (p < 0.05) but was not sustained at day 23 (p > 0.05); there were fewer Ki-67+ cells and more CC-3+ cells (p < 0.01).	No significant damage at ≤42 °C (H&E); risk of haemorrhage at higher energies noted.
29	Wu et al. [48]	Preclinical animal study	Canada	Sprague-Dawley rats with C6 glioma.	Rats: *n* = 37.	LIFU with a 1.06 MHz frequency.	LIFU (1.06 MHz, ISPTA ~ 5.5 W/cm², continuous wave, 20 min) with IV 5-ALA (60 mg/kg, 6 h prior); single or multi-point sonication targeting right cortex tumour; body temp 32 °C or 37 °C.	Reduced tumour growth (p < 0.01); extended survival (p < 0.01); multi-spot FUS improved survival vs. single-spot (p = 0.048); decreased proliferation, increased apoptosis (Ki-67, TUNEL).	No thermal lesions at 5.5 W/cm² (thermal dose <0.5 CEM43); minimal damage in normal brain (histology up to day 7).
30	Pi et al. [49]	Preclinical animal study	China	Balb/c nude mice with U87 MG-Red-FLuc GBM.	Mice: *n* = 36 (6 groups, *n* = 6 per group).	LIFU with a 1.0 MHz frequency.	Two-step LIFU: (1) BBB opening (1.0 MHz, 0.64 MPa, 10 ms pulses, 1 Hz, 60 s) with IV microbubbles and DVDMS (2 mg/kg). (2) SDT (1.0 MHz, 1.7 W, 300 ms pulses, 1 Hz, 60 s) 3 h later; repeated every 3 days ×3.	Enhanced DVDMS delivery (~3.4-fold); reduced tumour growth (BLI, p < 0.01); median survival 30.25 days vs. 23.75 days (p < 0.0001); increased apoptosis, decreased proliferation (IHC, p < 0.01).	No significant damage to normal tissue (histology at ~1 week); short durations and low intensities used.
31	Mainprize et al. [50]	Prospective, Phase I clinical trial. NCT02343991	Canada	Patients with primary high-grade glioma.	Patients: *n* = 5.	LIFU with a 220 kHz frequency.	LIFU (220 kHz, ~50% cavitation threshold, 50 s pulses, 0.74% duty cycle) with IV microbubbles (4 µL/kg per sonication) and chemo (doxorubicin or temozolomide); 2–5 spots targeting tumour margins; 1 day pre-resection.	BBB opening in all (T1-gadolinium); higher chemo levels in sonicated tissue (2/5 patients); no survival data.	No FUS-related oedema, haemorrhage, or deficits; mild scalp discomfort; 1 aborted due to back pain.
32	Idbaih et al. [51]	Prospective, Phase I/IIa clinical trial. NCT02253212	France	Patients with recurrent GBM.	Patients: *n* = 19.	LIFU with a 1.05 MHz frequency.	LIFU (1.05 MHz, 0.41–1.15 MPa, 25,000 cycles, 150–270 s) via implanted device with IV microbubbles (0.1 mL/kg) and carboplatin (AUC4–5); monthly up to 6 cycles targeting tumour area.	BBB opening in 52/65 procedures; PFS 3.45 mo, OS 10.0 mo overall; robust opening subgroup: PFS 4.11 mo, OS 12.94 mo.	No dose-limiting toxicities; 2 transient oedema cases (resolved); no haemorrhage or seizures; mild scalp pain.
33	Ha et al. [52]	Preclinical animal study	South Korea	BALB/c-nu mice with U87MG glioma.	Mice: *n* = 4.	LIFU with a 1 MHz frequency.	LIFU (1 MHz, 0.1 W/cm², 10% duty cycle, 5 min) with IV NIR-Alb NP-microbubble complex; whole-brain targeting, tumour in right hemisphere grown for 3 days.	Brain fluorescence was 1.5× higher with US (p < 0.05).	No haemorrhage, swelling, or injury at 0.1 W/cm², 10% duty cycle; higher intensities (>0.1 W/cm²) caused damage.
34	Park et al. [53]	Preclinical animal study	USA	Sprague-Dawley rats with 9 L gliosarcoma.	Rats: *n* = 21.	LIFU with a 690 kHz frequency.	LIFU (690 kHz, 0.72 MPa, 10 ms pulses, 1 Hz, 60 s) with IV microbubbles (10 µL/kg) and doxorubicin (5.67 mg/kg); 5 spots targeting bilateral tumours.	K^trans^ increased (p < 0.001); DOX concentration was higher in sonicated tissue (p < 0.01); correlation R² = 0.56.	Mild petechiae in sonicated areas; no large haemorrhage or major damage.
35	Lin et al. [54]	Preclinical animal study	China	Sprague-Dawley rats with C6 glioma.	Rats: *n* = 50.	LIFU with a 690 kHz frequency.	LIFU (690 kHz, MI = 1.9, 10 s pulses) with IV microbubbles (300 µL/kg) and DOX-cationic liposomes (5.67 mg/kg, 86% efficiency); twice weekly ×8 targeting right frontal lobe tumour.	Tumour volume is smallest with FUS + DOX-CLs (p < 0.001); median survival 81.2 days vs. 35 days (DOX-CLs) or 30.4 days (DOX); high apoptosis (TUNEL).	No organ toxicity (heart, liver, spleen, lung, kidney) at 17.01 mg/kg DOX-CLs after 1 month.
36	Carpentier et al. [55]	Prospective, Phase I/IIa clinical trial. NCT02253212.	France	Patients with recurrent GBM.	Patients: *n* = 15.	LIFU with a 1.05 MHz frequency.	LIFU (1.05 MHz, 0.5–1.1 MPa, 2.5 min, 2.38% duty cycle) via implanted device, with IV microbubbles (0.1 mL/kg), followed by carboplatin chemotherapy, applied monthly to the tumour/peritumoural region.	BBB opening in 28/41 sonications (≥0.8 MPa); 9/15 with robust opening had no local progression; 1 stable at 4 months.	No acute haemorrhage, ischaemia, or deficits; 1 transient oedema (tumour-related); mild scalp pain; 1 small unrelated stroke.
37	Yang et al. [56]	Preclinical animal study	Taiwan	Fischer 344 rats with F98/FGT glioma.	Rats: *n* = 36.	LIFU with a 1 MHz frequency.	LIFU (1 MHz, 0.7 MPa, 5% duty cycle, 60 s) with IV microbubbles; imaging (day 10), therapy (days 11, 14, 17) with GCV targeting the right hemisphere tumour.	Tumour growth reduced with FUS + GCV (p < 0.05); enhanced 123I-FIAU uptake (p < 0.05); survival prolonged vs. controls (<23 days).	No major AEs from FUS; no thermal damage or deficits reported.
38	Chen et al. [57]	Preclinical animal study	Taiwan	Sprague-Dawley rats with C6 glioma.	Rats: *n* = 48.	LIFU with a 0.5 MHz frequency.	LIFU (0.5 MHz, 0.36–0.7 MPa, 100 ms bursts, 1 Hz, 90 s) with IV microbubbles; IL-12 (0.3 µg/kg/day IP, days 11–15) targeting right striatum tumour.	IL-12 in the tumour: ~632 pg/mg vs. ~220 pg/mg control (p < 0.003); tumour growth suppressed; median survival 30 days vs. 20 days control (p < 0.001).	No red blood cell extravasation or T-cell changes at 5 W; higher power (20 W) caused petechiae.
39	Coluccia D et al. [58]	Phase I clinical case report	Switzerland	Patient with recurrent GBM.	Patient: *n* = 1.	HIFU with a 650 kHz frequency.	HIFU (650 kHz, 10–25 s pulses, up to 19,950 J) with MRI guidance; 25 sonications targeting left thalamic/subthalamic tumours.	~0.7 cc ablated (~10% of 6.5 cc tumour); stable ablation at 21 days; mild arm strength improvement.	No haemorrhage, oedema, or deficits; mild warmth sensation; no thermal injury to healthy tissue.
40	McDannold et al. [59]	Prospective, Phase I feasibility clinical trial (three patients). No NCT available.	USA	Patients with GBM.	Patients: *n* = 3.	HIFU with a 670 kHz frequency.	HIFU (670 kHz, 650–800 W, 20 s pulses) with MRI guidance; multiple sonications targeting thalamic tumours.	Maximal focal temperature 51 °C; no ablation achieved; no tumour changes observed.	No skin burns or pain (except 1 patient at 650 W); brain surface heating 0.9–3.8 °C.

*5-ALA* 5-aminolevulinic acid, *AEs* adverse events, *BBB* blood–brain barrier, *BLI* bioluminescence imaging, *BTB* blood–tumour barrier, *CAR T cells* chimeric antigen receptor T cells, *CI* confidence interval, *DCE-MRI* dynamic contrast-enhanced magnetic resonance imaging, *DOX* doxorubicin, *FUS* focused ultrasound, *GBM* glioblastoma, *GCV* ganciclovir, *Gy* Grey, *H&E* Haematoxylin and Eosin staining, *HIFU* high-intensity focused ultrasound, *IL-12* interleukin-12, *IP* intraperitoneal, *IV* intravenous, *LIFU* low-intensity focused ultrasound, *MB* microbubble, *MBs* microbubbles, *MGMT* O⁶-methylguanine-DNA methyltransferase, *MI* mechanical Index, *MRI* magnetic resonance imaging, *NIR-Alb* NP near-infra-red albumin nanoparticle, *OS* overall survival, *PD-1* programmed death-1, *PD-L1* programmed death-ligand 1, *PDX* patient-derived xenograft, *PFS* progression-free survival, *PRF* pulse repetition frequency, *RT* radiation therapy, *SDT* sonodynamic therapy, *SPIO* superparamagnetic iron oxide, *TAMs* tumour-associated macrophages, *TMZ* temozolomide.

### Risk of bias within studies

**Fig. 2:**
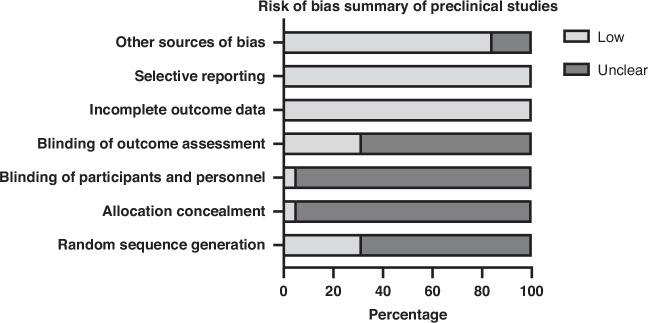
Summary of risk-of-bias assessments in preclinical studies using the SYRCLE tool. Each domain is presented as the percentage of studies rated as low or unclear risk of bias across the included preclinical studies (*n* = 26). Bars indicating low risk reflect adequate methodological reporting, whereas unclear risk indicates insufficient information to judge the domain. Common areas of uncertainty included sequence generation, allocation concealment, blinding of participants and personnel, blinding of outcome assessment, and incomplete outcome data. Image generated using Graphpad Prism. SYRCLE Systematic Review Centre for Laboratory Animal Experimentation.

**Fig. 3:**
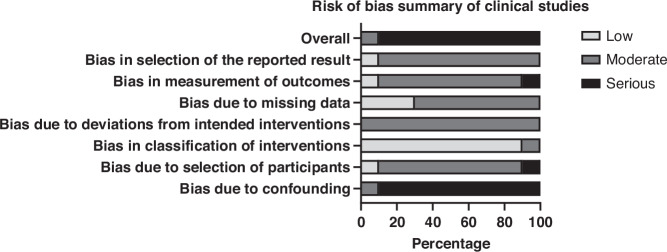
Summary of risk-of-bias assessments in clinical (non-randomised) studies using the ROBINS-I tool. Each domain is presented as the percentage of studies rated as low, moderate, or serious risk of bias across the included clinical studies (*n* = 14). Domains assessed include confounding, selection of participants, classification of interventions, deviations from intended interventions, missing data, measurement of outcomes, and selection of the reported result. Most studies demonstrated moderate to serious concerns across multiple domains, reflecting limitations typical of non-randomised designs. Image generated using Graphpad Prism. ROBINS-I Risk Of Bias In Non-randomised Studies of Interventions.

Bias in the 40 included studies was assessed using the SYRCLE tool for 26 preclinical studies [21, 23, 25, 26, 28–30, 32–35, 37, 39, 41, 42, 44–49, 52–54, 56, 57] and the ROBINS-I tool for 14 non-randomised clinical studies [20, 22, 24, 27, 31, 36, 38, 40, 43, 50, 51, 55, 58, 59], with results summarised in Figs. 2 and 3, respectively.

Preclinical studies showed ‘unclear’ risk in random sequence generation (62%), allocation concealment (85%), and blinding (96% personnel, 88% outcome assessors), but ‘low’ risk for incomplete outcome data and selective reporting (100%). Most studies (approximately 85–90%) demonstrated a low risk for other sources of bias, although a minority remained unclear due to limited reporting on conflicts of interest or experimental standardisation, underscoring the need for improved methodological transparency.

Clinical studies had ‘serious’ confounding bias (90%) due to small samples and inadequate controls, though intervention classification was ‘low’ risk (90%). Most other domains (e.g., participant selection, missing data) showed ‘moderate’ risk. Overall, 90% of clinical studies were rated ‘serious’ risk, with 10% ‘moderate’, highlighting challenges in non-randomised studies due to confounding and blinding issues.

### Synthesis of findings

This section synthesises the efficacy and safety evidence for LIFU and HIFU in the treatment of GBM, drawing from both preclinical and clinical studies. Due to the heterogeneity of the studies in terms of design, interventions, and outcomes, a narrative synthesis approach is adopted to summarise the findings. The section is organised to explore LIFU applications, including BBB opening (Section ‘Low-intensity focused ultrasound for blood-brain barrier opening’) and SDT (Section ‘Low-intensity focused ultrasound in sonodynamic therapy’), followed by HIFU (Section ‘High-intensity focused ultrasound for thermal ablation and therapeutic delivery’), and a comparison of these modalities in the context of GBM treatment.

#### Low-intensity focused ultrasound for blood-brain barrier opening

LIFU enhances drug delivery in GBM by transiently and non-invasively opening the BBB using intravenous microbubbles. This section reviews preclinical and clinical evidence of its efficacy and safety.

#### Preclinical studies: efficacy and safety

Preclinical models show that LIFU significantly enhances delivery of chemotherapies, immunotherapies, gene therapies, and nanoparticles, resulting in reduced tumour growth and extended survival. LIFU increased brain concentrations of drugs like doxorubicin, etoposide, and cetuximab, prolonging survival (e.g., 18–30.4 days to 26–81.2 days with doxorubicin [39, 53, 54]. Similarly, etoposide delivery increased 8-fold, reducing tumour burden by ~45% and survival from 19 to 25 days [34]. Cetuximab improved survival from 34.90 to 39.82 days [28]. Irinotecan, however, showed inconsistent survival benefits despite BBB opening [44].

LIFU also enhances immunotherapy delivery, including immune checkpoint inhibitors, CAR T cell numbers, and cytokines such as interferons, interleukins, and GM-CSF [60], boosting anti-tumour immunity. These effects are particularly promising in the context of GBM’s immune resistance, where tumour-associated macrophages dominate the immune infiltrate and inhibit cytotoxic T cell responses [61]. GBM tumours often express immune checkpoint ligands like Programmed Death-Ligand 1(PD-L1) [62, 63] but exhibit poor infiltration by CD8 + T cells, contributing to a non-inflamed, immunologically ‘cold’ microenvironment [64]. Enhancing immune cell trafficking and activation via LIFU could be critical for overcoming these barriers. Anti-PD-1 antibodies with LIFU increased survival, achieving remission in some models with greater immune cell infiltration [24, 32]. CAR T cells with LIFU extended survival to 80 days versus 35 days [35]. IL-12 delivery raised deposition levels of the cytokine in the brain, extending survival from 20 to 30 days [57]. Additionally, radiolabelled anti-PD-L1 antibodies enabled tumour imaging with improved uptake, underscoring LIFU’s potential in diagnostic applications [30]. LIFU alone also modulates the tumour microenvironment, increasing pro-inflammatory TAMs, microglia, and T-cell infiltration, suggesting intrinsic immunomodulatory effects [25, 40].

Gene therapies and nanoparticle-based approaches further highlight LIFU’s efficacy. Delivery of oncolytic viruses and CRISPR/Cas9 nanoparticles reduced tumour growth and restored chemosensitivity, with survival extended by up to 50% or from 22–30 days to 43 days in treated groups [23, 33]. Gene therapy with ganciclovir also suppressed tumour progression, prolonging survival beyond 23 days [56]. Nanoparticle delivery increased transgene expression (~4-fold), improved transfection volumes, and extended median survival (28–30.25 days vs. 23.75–24) [26, 41, 45, 52]. Topotecan delivery was similarly enhanced, supporting LIFU’s broad applicability [37]. In radiolabelled antibody studies, LIFU improved tumour targeting in patient-derived xenografts, further expanding its therapeutic scope [46].

Combination therapies, including radiotherapy and sonodynamic therapy, amplify LIFU’s effects. When paired with radiation, LIFU reduced tumour volumes by 45–57% and improved survival, demonstrating synergy [21, 31]. LIFU has also enhanced sonosensitiser delivery for SDT, improving therapeutic outcomes in preclinical GBM models [48, 49], as discussed in detail in Section ‘Low-intensity focused ultrasound in sonodynamic therapy’. LIFU with biomimetic microbubbles and post-radiation settings also enhanced drug delivery and BBB opening, reinforcing its combinatorial potential [42].

Safety is favourable, with transient BBB opening resolving within hours to days and no long-term damage [23, 25, 28, 40, 42]. Mild, resolving petechiae or microhaemorrhages occur rarely, with no neurological deficits [21, 34, 44, 53]. Optimised parameters ensure stable cavitation, minimising risks [32, 44]. No significant toxicity, weight loss, or histological damage is reported [24, 26, 30, 33, 35, 37, 39, 41, 45, 46, 48, 49, 52, 54, 56, 57].

#### Clinical studies: efficacy and safety

Early-phase clinical trials demonstrate the feasibility and safety of LIFU in GBM, with preliminary signs of efficacy. BBB opening is reliably achieved, facilitating delivery of agents like temozolomide, carboplatin, doxorubicin, and albumin-bound paclitaxel [20, 22, 24, 27, 40, 43, 50, 51, 55]. For instance, robust BBB opening was achieved in 90% of sonication targets in a cohort of 33 patients, correlating with higher carboplatin levels [22]. Similarly, doxorubicin and anti-PD-1 concentrations doubled in treated areas, suggesting improved therapeutic access [24]. LIFU with an implantable ultrasound device also enhanced the delivery of albumin-bound paclitaxel in recurrent GBM patients, achieving greater drug penetration into the tumour [27]. In another trial, LIFU with temozolomide resulted in 100% one-year survival among six patients, with four showing no recurrence at follow-up [43].

Survival outcomes, while limited by small sample sizes, indicate potential benefits. LIFU with carboplatin yielded progression-free survival (PFS) of 2.5–4.11 months and overall survival (OS) of 10.0–14.0 months, with subgroups experiencing robust BBB opening showing superior outcomes [22, 51]. In recurrent GBM, LIFU prevented local progression in some patients, with one maintaining stability at four months [55]. When combined with radiotherapy, LIFU achieved stable disease in half of a small cohort for over five months, with a median PFS of approximately 97.5 days [31]. These findings suggest LIFU may enhance treatment efficacy, though larger trials are needed to confirm these trends.

Clinical safety is well-established. BBB opening is reversible within 24 h, with no severe FUS-related events [20, 40, 43]. Mild, reversible side effects, including scalp pain, nausea, headache, and dizziness, are common but resolve quickly [22, 24, 50, 51, 55]. Rare instances of transient oedema or pseudoprogression occur but do not require intervention [43, 51]. Minimal petechiae are occasionally observed, with no evidence of haemorrhage, ischaemia, or long-term neurological deficits [20, 31]. Moreover, multiple sessions of LIFU-induced BBB opening with temozolomide were shown to be safe and feasible, with no severe adverse events reported across repeated treatments [36]. Additionally, LIFU was safely employed to enhance the release of circulating biomarkers in brain tumour patients, further supporting the tolerability of BBB opening in clinical settings [38]. These outcomes underscore LIFU’s safety for repeated applications, even in complex cases involving implantable devices or combination therapies.

#### Low-intensity focused ultrasound in sonodynamic therapy

SDT leverages LIFU to activate sonosensitisers, generating reactive oxygen species that induce tumour cell death in GBM. Importantly, the current evidence for SDT in GBM is derived exclusively from preclinical studies; no clinical trials meeting our inclusion criteria were identified or excluded during the literature selection process. The following section reviews these preclinical investigations, which explore LIFU’s role in SDT either by enhancing sonosensitiser delivery through BBB opening or by directly activating sonosensitisers for cytotoxic effects.

#### Preclinical studies: efficacy and safety

Preclinical investigations have demonstrated that LIFU can support SDT either by enhancing sonosensitiser delivery through BBB opening or by directly activating sonosensitisers to induce tumour cytotoxicity. In one study, LIFU with microbubbles facilitated SDT with 5-aminolevulinic acid (5-ALA) in a rat glioma model, enhancing BBB permeability to improve sonosensitiser uptake [48]. This approach reduced tumour proliferation and significantly extended survival compared to controls. Another study used LIFU with microbubbles to deliver sinoporphyrin sodium for SDT in a GBM xenograft model, achieving enhanced BBB opening and improved therapeutic outcomes, with multi-spot sonication outperforming single-spot approaches [49].

In contrast, a study employing 220-kHz LIFU with 5-ALA induced direct cytotoxic effects in a glioma model, focusing on sonosensitiser activation rather than BBB opening [47]. This approach reported reduced tumour growth, highlighting SDT’s potential as a standalone LIFU application. Unlike the studies leveraging BBB opening for sonosensitiser delivery [48, 49], the latter study primarily utilised LIFU for direct tumour cytotoxicity, underscoring SDT’s versatility.

These findings suggest LIFU’s promise in SDT, with applications ranging from BBB-mediated delivery to direct tumour cytotoxicity. The favourable safety profile observed in these studies, with no significant reported toxicity, supports further investigation. However, translation to the clinic will require optimisation of ultrasound parameters and confirmation of efficacy and safety in human trials.

#### High-intensity focused ultrasound for thermal ablation and therapeutic delivery

HIFU induces thermal ablation by heating tissue above 60 °C, causing tumour cell destruction, and enhances drug delivery via cavitation with microbubbles or nanoparticles. This section reviews HIFU’s efficacy and safety in GBM treatment.

#### Preclinical studies: efficacy and safety

Preclinical studies demonstrate that HIFU reduces tumour growth and enhances drug delivery in GBM models. In GL261 glioma-bearing mice, HIFU (1.1 MHz) combined with doxorubicin-loaded microbubbles and magnetic targeting significantly reduced tumour growth (*p* < 0.05) and increased drug accumulation (*p* < 0.01), with decreased cellular proliferation and increased apoptosis [29]. No brain or organ damage was observed. In another study, HIFU (1.5 MHz) with radiotracers increased topotecan uptake (~2.1% vs. 0.88% ID/g, *p* < 0.05) and enabled BBB opening without causing haemorrhage [37]. In NOD-SCID mice, HIFU (7 MHz) in combination with nanobubbles achieved approximately 70% tumour growth inhibition, improved survival (*p* < 0.05), and enhanced MRI contrast, with no observed toxicity or off-target effects [41]. These findings support the efficacy of HIFU in reducing tumour burden and enhancing therapeutic delivery, with a favourable safety profile when parameters are appropriately optimised.

#### Clinical studies: efficacy and safety

Clinical HIFU data are limited to early-phase trials. A phase I case report used HIFU (650 kHz) with MRI guidance for recurrent GBM, ablating ~10% of a thalamic tumour, with stable results at 21 days and mild arm strength improvement [58]. No haemorrhage or neurological deficits occurred, only a mild warm sensation. A phase I trial in three GBM patients (670 kHz, 650–800 W) reached 51 °C but achieved no ablation, with minimal brain surface heating (0.9–3.8  °C) and no severe adverse events, except pain in one patient [59]. These clinical studies indicate that HIFU is feasible and safe for GBM treatment, with no severe adverse events reported. However, efficacy outcomes are limited, with only partial ablation achieved in one case and no tumour response in another. The small sample sizes and early-phase nature of these trials underscore the need for further optimisation and larger studies to establish HIFU’s therapeutic potential. Furthermore, the efficacy of thermal ablation is constrained by GBM’s infiltrative borders and heterogenous cellular composition, where tumour cells can extend beyond contrast-enhancing regions and evade localised treatment [65]. This underscores the importance of combinatory strategies that address both core and invasive margins.

#### Comparison of LIFU and HIFU in GBM treatment

LIFU and HIFU offer distinct approaches to GBM treatment. LIFU enhances drug delivery by transiently opening the BBB, while HIFU induces thermal ablation to destroy tumour tissue. This section compares their mechanisms, efficacy, safety, and clinical roles, with Table 2 summarising key differences and synergies.

**Table 2:** Comparison of LIFU and HIFU for GBM treatment: mechanisms, efficacy, safety, and clinical applications.

Aspect	LIFU	HIFU
Primary mechanism	Stable cavitation of microbubbles to transiently open BBB, enhancing drug delivery [11, 12].	Thermal ablation via rapid tissue heating (>60 °C) inducing coagulative necrosis; can also enhance drug delivery via cavitation [14, 15].
Frequency range	Typically 0.2–1.5 MHz [22, 28, 34].	Typically 0.65–7 MHz [29, 41, 58, 59].
Preclinical efficacy	Reduced tumour growth (e.g., 45% reduction with etoposide); prolonged survival (e.g., 26–81.2 days vs. 18–30.4 days); enhanced immune responses [24, 34, 39, 53, 54].	~70% tumour growth inhibition; improved survival (*p* < 0.05); increased drug uptake (e.g., 2.1% ID/g topotecan) [29, 37, 41].
Clinical efficacy	PFS 2.5–4.11 months, OS 10–14 months with carboplatin; 100% 1-year survival with temozolomide in small cohorts [22, 43, 51].	Partial ablation (~10% of the tumour) in one case; no tumour response in another; limited by small sample sizes [58, 59].
Safety profile	Transient, mild effects (e.g., petechiae, scalp pain, nausea); BBB closes within 24 h; no long-term deficits [20, 22, 40].	No significant toxicity or haemorrhage; mild effects (e.g., warmth, minor pain); risk of unintended heating [29, 37, 41, 58, 59].
Adverse events	Mild, reversible (e.g., headache, dizziness); rare transient oedema or pseudoprogression [43, 51].	Mild, reversible (e.g., warmth sensation, pain in one case); no severe events reported [58, 59].
Clinical applications	Enhances delivery of chemotherapies, immunotherapies, and gene therapies; suitable for recurrent GBM and combination therapies [22, 31].	Localised tumour debulking is best for superficial tumours and has potential for drug delivery enhancement [29, 58].
Challenges	Variability in drug-specific outcomes; need for larger clinical trials to confirm survival benefits [44].	Skull attenuation, limited penetration depth, suboptimal ablation in deep tumours, and early-phase trial limitations [59].
Synergistic potential	Can enhance drug delivery prior to other therapies; combinable with radiotherapy or SDT [21, 31, 48].	Can be preceded by LIFU for enhanced drug penetration; combinable with targeted agents for dual effects [29, 37].

*BBB* Blood-Brain Barrier, *HIFU* High-Intensity Focused Ultrasound, *ID/g* Injected Dose per gram, *LIFU* Low-Intensity Focused Ultrasound, *OS* Overall Survival, *PFS* Progression-Free Survival, *SDT* Sonodynamic Therapy.

LIFU uses stable cavitation of microbubbles to open BBB tight junctions, improving delivery of chemotherapies, immunotherapies, and nanoparticles [11, 12]. HIFU, conversely, employs high-energy waves to heat tissue above 60 °C, causing coagulative necrosis, and can enhance drug delivery via cavitation [14, 15, 29, 37, 41]. Preclinically, LIFU reduces tumour growth and extends survival (e.g., 26–81.2 days vs. 18–30.4 days) [24, 34, 39, 53, 54], while clinical trials show promising survival (e.g., 100% 1-year survival with temozolomide) [22, 43, 51]. HIFU achieves ~70% tumour growth inhibition preclinically but has limited clinical efficacy, with only partial ablation (~10%) in one case [29, 41, 58, 59].

Both modalities are safe, with LIFU causing transient effects (e.g., petechiae, nausea) and HIFU showing mild, reversible effects (e.g., warmth) [20, 22, 29, 37, 40, 58, 59]. HIFU’s thermal risks require precise MRI guidance [59]. LIFU suits combination therapies for recurrent GBM, while HIFU is ideal for localised debulking but is limited by skull attenuation [22, 31, 59].

### Ultrasound parameters and outcomes

The included studies used a wide range of ultrasound parameters, contributing to variability in outcomes as shown in Table 3. LIFU frequencies ranged from 0.22 to 1.64 MHz, with intensities of 0.3–1.03 MPa or MI 0.4–1.9 (varying units across studies) and durations from 10 s to 4.5 min. Preclinical LIFU studies reported enhanced drug delivery (e.g., 8-fold etoposide increase [34]) and survival benefits (e.g., 26–81.2 days across studies like [39, 54]). Clinical LIFU trials achieved consistent BBB opening and improved survival (e.g., 100% one-year survival [43]). HIFU frequencies spanned 650 kHz to 7 MHz, with intensities up to 800 W or 0.7 MPa, and durations from 10 ms bursts to 4 min. Preclinical HIFU studies demonstrated ~70% tumour growth inhibition [29, 41], while clinical trials achieved limited ablation (~10% [58]) or none [59]. These findings underscore the need for standardised parameters to optimise FUS efficacy.

**Table 3:** Ultrasound parameters and efficacy outcomes for 40 studies on FUS in GBM.

Study ID	FUS modality	Frequency (MHz)	Intensity	Duration	Efficacy outcomes
McDannold et al. [20]	LIFU	0.22	NR	38 sessions	BBB opening; stable disease in 5/9 patients
Fletcher et al. [21]	LIFU	0.22	~1.1 Hz PRF	5 ms bursts	45–57% tumour volume reduction; increased survival
Carpentier et al. [22]	LIFU	1.0	1.03 MPa	270 s	PFS 2.5–3.1 mo, OS 12–14 mo; 90% BBB opening
Bhoopathi et al. [23]	LIFU	1.0	3.5 mV, 10 dB	60 s	~50% longer survival; reduced tumour growth
Arrieta et al. [24]	LIFU	1.0	~0.3 MPa	60 s	2–6-fold drug uptake; prolonged survival
Zhang et al. [25]	LIFU	1.5	~0.5 MPa	NR	Increased TAMs/monocytes; no survival data
Sun et al. [26]	LIFU	0.69	0.32 MPa	100 s	Median survival 28 vs. 24 days; tumour suppression
Sonabend et al. [27]	LIFU	1.0	1.03 MPa	4.5 min	3.7-fold paclitaxel uptake; PFS 2.9 mo, OS 11 mo
Porret et al. [28]	LIFU	1.5	0.43 MPa	127 s	Mean survival 39.82 vs 34.90 days
He et al. [29]	HIFU	1.1	1 V	4 min/session	Reduced tumour growth; increased apoptosis
Chevaleyre et al. [30]	LIFU	1.5	0.42 MPa	127 s	Increased PD-L1 uptake; no survival data
Chen et al. [31]	LIFU	0.5	MI 0.4–0.68	120 s	Preclinical: improved survival; Clinical: PFS ~ 97.5 days
Lee et al. [32]	LIFU	1.64	NR	~2 min/target	Prolonged survival; 2.5-fold anti-PD1 uptake
Yang et al. [33]	LIFU	NR	1.84 W	3–5 min	Median survival 43 vs. 22–30 days
Wei et al. [34]	LIFU	1.5	0.7 MPa	NR	8-fold etoposide uptake; median survival 25 vs. 19 days
Sabbagh et al. [35]	LIFU	1.0	0.3 MPa	120 s	Survival 34–80 vs. 24–39 days
Park et al. [36]	LIFU	0.22	NR	~90 s	92.4% BBB opening; stable disease in 5/6 patients
Molotkov et al. [37]	HIFU	1.5	0.7 MPa	10 ms bursts	2.1-fold topotecan uptake; no survival data
Meng et al. [38]	LIFU	0.22	NR	111 ± 39 min	2.6–3.2-fold biomarker increase; no survival data
Liao et al. [39]	SW	NR	~0.21 mJ/mm²	~40 s	Median survival 26 vs. 18 days; reduced tumour size
Chen et al. [40]	LIFU	0.5	MI 0.48–0.81	120 s	Dose-dependent BBB opening; increased T-cell infiltration
Chan et al. [41]	HIFU	7.0	NR	NR	~70% tumour inhibition; improved survival
Wang et al. [42]	LIFU	0.4–0.6	0.72 MPa	NR	Enhanced Gadolinium uptake; no survival data
Park et al. [43]	LIFU	0.22	NR	~210 s/target	100% 1-year survival; 4/6 no recurrence
McDannold et al. [44]	LIFU	0.23	NR	55 s	Increased irinotecan uptake; no survival benefit
Curley et al. [45]	LIFU	1.1	0.45–0.55 MPa	2 min/spot	4-fold transgene expression; no survival data
Brighi et al. [46]	LIFU	1.1	0.85 MPa	120 s/spot	Increased antibody uptake; no survival data
Yoshida et al. [47]	LIFU	0.22	18 W	30 s × 10	Reduced tumour growth (day 16); not sustained
Wu et al. [48]	LIFU	1.06	~5.5 W/cm²	20 min	Extended survival; reduced tumour growth
Pi et al. [49]	LIFU	1.0	0.64 MPa (BBB), 1.7 W (SDT)	60 s	Median survival: 30.25 vs. 23.75 days
Mainprize et al. [50]	LIFU	0.22	NR	50 s	Higher chemo in sonicated tissue; no survival data
Idbaih et al. [51]	LIFU	1.05	0.41–1.15 MPa	150–270 s	PFS 3.45–4.11 mo, OS 10–12.94 mo
Ha et al. [52]	LIFU	1.0	0.1 W/cm²	5 min	1.5-fold fluorescence; no survival data
Park et al. [53]	LIFU	0.69	0.72 MPa	60 s	Higher doxorubicin uptake; no survival data
Lin et al. [54]	LIFU	0.69	MI 1.9	10 s	Median survival: 81.2 vs. 30.4 days
Carpentier et al. [55]	LIFU	1.05	0.5–1.1 MPa	2.5 min	BBB opening in 28/41 sonications; stable at 4 mo
Yang et al. [56]	LIFU	1.0	0.7 MPa	60 s	Prolonged survival; reduced tumour growth
Chen et al. [57]	LIFU	0.5	0.36–0.7 MPa	90 s	Median survival: 30 vs. 20 days
Coluccia D et al. [58]	HIFU	0.65	NR	10–25 s	~10% tumour ablation; stable at 21 days
McDannold et al. [59]	HIFU	0.67	650–800 W	20 s	No ablation; no tumour changes.

*BBB* blood-brain barrier, *mo* months, *MPa* megapascals, *MI* mechanical index, *NR* not reported, *OS* overall survival, *PFS* progression-free survival, *PRF* pulse repetition frequency, *SDT* sonodynamic therapy, *SW* shockwave, *TAMs* tumour-associated macrophages.

## Discussion

Despite therapeutic advances, GBM remains one of the most treatment-resistant cancers, owing to its complex tumour biology. One of the central challenges is the extreme heterogeneity of GBM at both the cellular and molecular levels. Tumour cells exist in multiple, interconverting transcriptional states within a single lesion, complicating efforts to uniformly target the neoplastic population. Moreover, GBM co-opts a range of non-neoplastic cells which interact with tumour cells to promote survival, invasion, and resistance [66].

The immune microenvironment of GBM further limits therapeutic efficacy. Characterised as immunologically ‘cold,’ GBM is marked by low T cell infiltration, impaired antigen presentation, and a dominance of immunosuppressive tumour-associated macrophages (TAMs) that blunt anti-tumour immunity. This environment contributes to the failure of immune checkpoint inhibitors [67] and underscores the need for strategies that can both enhance drug delivery and reprogramme the immune landscape.

Compounding these challenges is the diffuse, infiltrative growth pattern of GBM. Tumour cells extend beyond the radiographic margins along white matter tracts and perivascular spaces [68], evading both surgical resection and focal therapies. The BBB, while compromised in the tumour core, remains largely intact in the peritumoural region, limiting drug penetration where it may be most needed [69]. Together, these biological barriers—heterogeneity, immune exclusion, diffuse invasion, and BBB integrity—necessitate combinatorial approaches.

A lot of focus still remains on understanding the mechanism of action of FUS in its ability to kill GBM cells and whether specific sonosensitizers play a role. While the induction of cell death is reported to be through reactive oxygen species generation and consequent apoptosis of the tumour cells [70], the overarching mechanism still remains debated in the field. The role of the immune system is still enigmatic, as SDT treatment has been reported to drive abscopal effects in animal models [71]. Hence, further basic research is needed to fully understand how SDT kills cancer.

This systematic review synthesises preclinical and clinical evidence on the efficacy and safety of LIFU and HIFU in GBM treatment, highlighting their potential to address critical therapeutic challenges. LIFU enhances drug delivery by transiently opening the BBB, showing reduced tumour growth and prolonged survival in preclinical models (e.g., 26–81.2 vs. 18–30.4 days) [39, 54] and promising clinical outcomes (e.g., 100% one-year survival with temozolomide) [43]. HIFU, conversely, provides thermal ablation and supports therapeutic delivery, with preclinical studies reporting ~70% tumour inhibition, although clinical efficacy remains limited (e.g., ~10% ablation in one case) [41, 58]. Both modalities exhibit favourable safety profiles, with mild, transient adverse effects (e.g., petechiae for LIFU, warmth for HIFU) and no long-term deficits [20, 22, 37, 58].

LIFU’s ability to enhance chemotherapies, immunotherapies, and gene therapies makes it a versatile adjunct to standard treatments, particularly for recurrent GBM where the BBB remains intact. Its non-invasive, repeatable nature facilitates ongoing therapeutic access [22, 24, 33]. HIFU complements LIFU through localised tumour debulking, although its effectiveness is limited by technical barriers such as skull attenuation [59]. Microbubble-enhanced HIFU has shown combined drug delivery and tumour inhibition (~70%) in GBM xenografts [29, 37], hypothesising that sequential LIFU for drug delivery followed by HIFU for ablation could maximise efficacy, pending further preclinical validation.

Several technical variables influence FUS efficacy and safety, notably the physicochemical design of microbubbles and the configuration of ultrasound devices. The efficacy of FUS in GBM, particularly for LIFU-mediated BBB opening, is strongly influenced by microbubble composition, which determines cavitation efficiency, circulation time, and safety. Across the included studies, microbubble formulations varied markedly; differences in shell material, elasticity, and gas content altered acoustic behaviour and therapeutic outcomes. In clinical research, the most frequently used agents were the commercially available Definity®, containing a lipid shell and perflutren (C₃F₈) gas [20, 38, 50]; SonoVue®, composed of a phospholipid shell and sulphur hexafluoride (SF₆) [22, 51, 55]; and Optison®, which uses an albumin shell with perfluoropropane (C₃F₈) [36, 43]. These agents were selected for their regulatory approval and predictable pharmacokinetics. The flexibility and low acoustic impedance of lipid shells facilitated stable cavitation at pressures of roughly 0.3–1.0 MPa, achieving consistent BBB opening in up to 90% of sonication targets in one trial [22] while minimising adverse effects such as transient petechiae or mild scalp discomfort [20, 22, 43]. Gases with low diffusivity, including perfluorocarbons and SF₆, prolonged bubble stability during sonication, enabling repeated BBB-opening sessions in recurrent GBM [36].

Building on these clinically validated formulations and their established safety profiles, preclinical studies have developed customised and multifunctional microbubbles to enhance delivery efficiency and targeting precision. Lipid-shelled bubbles filled with perfluorobutane or perfluoropropane were common [34, 45, 54], achieving up to an eightfold increase in etoposide concentration [34] and prolonging survival from 26 to 81 days [39, 54]. Innovative designs such as cytomembrane-coated microbubbles for magnetic targeting [29] or PEG-coated polymer shells for gene delivery [26, 52] further improved tumour specificity and transfection efficiency. Some nanobubble formulations achieved about 70% tumour-growth inhibition under HIFU [41]. Flexible lipid shells generally outperformed rigid protein or polymer shells in terms of BBB-opening volume, while perfluorocarbon gases supported more sustained cavitation than air-filled counterparts. Although most studies reported no structural or histological toxicity, several noted brief, localised glial activation after BBB opening [28, 30], consistent with transient neuroinflammatory responses rather than tissue injury. Collectively, these findings highlight the need to optimise microbubble biocompatibility and acoustic dosing. Lipid-shelled bubbles filled with perfluorocarbon or SF₆ gases currently offer the best balance between efficacy and safety, whereas emerging biomimetic and multifunctional designs [29] show promise for GBM-specific targeting and controlled drug release.

Beyond microbubble formulation, the architecture of the ultrasound device itself strongly influences treatment precision, penetration depth, and repeatability. Device configuration, particularly the distinction between externally applied, MRI-guided phased-array systems and implantable emitters, affects focal accuracy, energy deposition, and therapeutic reproducibility. Externally applied hemispherical phased-array transducers operating at 220 kHz to 1.5 MHz were used in both preclinical and clinical studies [20, 21, 38, 44, 50], enabling volumetric targeting through electronic beam steering and integration with MRI guidance. These non-invasive systems are scalable across centres and allow repeated treatments without surgery, but skull attenuation and acoustic aberrations can compromise energy transmission, especially in patients with irregular bone structures or resection cavities [59]. In HIFU applications, phased arrays operate at higher frequencies (650 kHz to 7 MHz) and powers approaching 800 W [59], requiring precise MRI guidance to maintain thermal confinement and avoid off-target heating.

In contrast, implantable ultrasound emitters such as the nine-emitter SonoCloud device, evaluated in trials NCT02253212, NCT03744026 and NCT04528680 [22, 24, 27, 51, 55], bypass skull-related distortions and achieve consistent BBB opening with lower acoustic energy, supporting repeated localised delivery in recurrent GBM [22, 27, 55]. Nevertheless, implantation requires craniotomy and carries surgical risks such as infection or fibrosis at the device interface, and the treatment zone is limited to the emitter array. Continued standardisation of device geometry, element configuration, and acoustic calibration, together with transparent reporting of treatment planning and dosimetry, will be critical to refine safety thresholds and ensure reproducibility across clinical centres.

Compared to other GBM therapies, FUS offers distinct advantages. Immunotherapies face systemic toxicity and limited tumour infiltration, whereas LIFU improves delivery of agents like checkpoint inhibitors and CAR T cells with minimal toxicity [24, 35]. Tumour-treating fields are non-invasive but demand prolonged use and yield modest survival (~20.9 months), compared to LIFU’s early clinical promise [72]. Targeted therapies, such as bevacizumab, are constrained by BBB penetration, which LIFU can overcome [73]. However, FUS adoption is hindered by equipment costs, the need for specialised training, and limited accessibility compared to conventional pharmacotherapies.

Beyond GBM, clinical experience with FUS in extracranial tumours underscores its translatability and scalability. In localised prostate cancer, the prospective multicentre HIFI trial (*n* = 3328; 46 centres) demonstrated that whole-gland or subtotal HIFU achieved non-inferior 30-month salvage therapy–free survival compared to radical prostatectomy (hazard ratio 0.71, 95% CI 0.52–0.97; p = 0.008), with significantly fewer adverse effects on urinary continence (29% vs 44%) and erectile function (mean score decline: –7 vs –13) [74]. These real-world data show that ablative FUS can deliver comparable cancer control to surgery with improved functional outcomes and that multicentre clinical protocols are feasible.

In breast cancer, LIFU has been used to enhance radiosensitivity through microbubble stimulation. A Phase I MR-guided FUS-microbubble trial reported a 50% complete response rate at 3 months and 88% local control at 12 months across 18 tumours, with only low-grade dermatitis as toxicity [75]. An earlier Phase I study similarly observed complete response in all evaluable lesions (7/7) at 3 months [76]. These studies illustrate that non-invasive FUS can be safely combined with standard therapies to enhance tumour control while minimising toxicity, principles that are directly relevant to GBM, particularly when designing multicentre, function-preserving treatment trials.

Clinical translation faces challenges: high equipment costs, limited MRI-guided FUS availability, and lack of standardised parameters. HIFU trials in particular are limited by small sample sizes and suboptimal ablation, necessitating larger, randomised clinical trials (RCTs) [58, 59]. Patient selection is critical, as tumour location, size, and prior treatments affect outcomes. Superficial tumours may benefit more from HIFU, while deep-seated or infiltrative GBMs are better suited to LIFU. Molecular features such as isocitrate dehydrogenase (IDH) gene mutations and O [6]-methylguanine-DNA methyltransferase (MGMT) promoter methylation may also influence therapy response and should guide future stratification.

This review has several limitations. First, significant heterogeneity among included studies, in terms of study design, animal models, FUS parameters, and outcome measures, limited comparability and precluded meta-analysis. For example, preclinical models varied (e.g., GL261, U87, F98), and FUS settings ranged widely (250 kHz–7 MHz; 0.5 MPa–800 W/cm²), while clinical studies differed in tumour stage and concurrent treatments. Second, quality assessments revealed notable bias. In preclinical studies, the SYRCLE tool indicated an ‘unclear’ risk in randomisation (62%), allocation concealment (85%), and blinding (96% for personnel, 88% for outcome assessors). Clinical studies assessed with ROBINS-I showed a ‘serious’ risk of bias (90%), driven by confounders, small cohorts, and a lack of control factors likely to inflate treatment effects. Third, translating preclinical results to clinical settings is difficult due to physiological and biological differences between animal models and human GBM. Rodent survival gains (e.g., 81.2 vs. 30.4 days) may not extrapolate directly to patients with more heterogeneous, infiltrative disease. Fourth, all included clinical studies were conducted in high-income countries with advanced healthcare infrastructure. This geographic concentration reflects strong early translational engagement, but it limits the generalisability of findings to lower-resource settings. The absence of studies from low- and middle-income countries (LMICs) may constrain the external validity of FUS applications, especially in health systems with different resource availability, patient demographics, or regulatory environments.

These limitations call for cautious interpretation and underscore the need for future research using standardised, rigorous methodologies. Key research priorities include:

1. Large, multicentre RCTs to confirm the efficacy and safety of LIFU and HIFU, alone or combined with standard therapies. Trials should stratify patients by tumour location, molecular subtype, and prior treatments, and report long-term outcomes such as PFS and OS. Importantly, future studies should also include sites across diverse geopolitical and healthcare settings, including LMICs, to evaluate real-world feasibility, cost-effectiveness, and equitable scalability of FUS technologies.
2. Parameter optimisation, especially for HIFU, to enhance ablation efficiency and reduce skull attenuation effects. This includes real-time acoustic feedback, refined frequency (650–1500 kHz), intensity (0.5–800 W/cm²), pulse duration, and novel transducer designs.
3. Integrated LIFU-HIFU protocols to leverage synergistic effects, with preclinical models exploring sequential or concurrent use alongside agents like immune checkpoint inhibitors or oncolytic viruses. Feasibility and dosing should then be tested in early-phase trials.
4. Cost-effectiveness analyses to assess FUS viability across health systems, accounting for equipment, training, and potential reductions in drug dosage. Trials should also incorporate patient-specific variables (e.g., BBB integrity, IDH/MGMT status, immune landscape) to guide personalised therapy. Advanced imaging and biomarker profiling (e.g., DCE-MRI) should be embedded into study design to refine patient selection.

## Conclusions

This systematic review suggests that LIFU and HIFU hold promise as adjunctive therapies for GBM. Preclinical data show that LIFU improves drug delivery and prolongs survival (e.g., 26–81.2 vs. 18–30.4 days), with early clinical trials reporting encouraging outcomes, including 100% one-year survival in a small cohort treated with LIFU and temozolomide. HIFU demonstrates substantial tumour inhibition (~70%) in preclinical studies, but clinical success is limited, with only partial or no ablation reported. Both modalities appear safe, producing only mild, transient side effects.

However, these findings must be interpreted cautiously due to substantial heterogeneity, high risks of bias, and the difficulty of translating preclinical results to humans. While the clinical promise of LIFU is emerging, evidence for HIFU remains sparse and inconclusive. Neither modality can yet be considered an established treatment for GBM. Rigorous, multicentre randomised trials are needed to confirm efficacy, standardise parameters, and overcome technical barriers, particularly for HIFU. Future research should also examine patient-specific factors to optimise outcomes.
